# Studying Autism in Rodent Models: Reconciling Endophenotypes with Comorbidities

**DOI:** 10.3389/fnhum.2013.00417

**Published:** 2013-07-25

**Authors:** Andrew Argyropoulos, Krista L. Gilby, Elisa L. Hill-Yardin

**Affiliations:** ^1^Department of Medicine, The University of Melbourne, Parkville, VIC, Australia; ^2^Department of Physiology, The University of Melbourne, Parkville, VIC, Australia

**Keywords:** autism, epilepsy, sleep, motor deficits, aggression, sensory, gastrointestinal function, anxiety

## Abstract

Autism spectrum disorder (ASD) patients commonly exhibit a variety of comorbid traits including seizures, anxiety, aggressive behavior, gastrointestinal problems, motor deficits, abnormal sensory processing, and sleep disturbances for which the cause is unknown. These features impact negatively on daily life and can exaggerate the effects of the core diagnostic traits (social communication deficits and repetitive behaviors). Studying endophenotypes relevant to both core and comorbid features of ASD in rodent models can provide insight into biological mechanisms underlying these disorders. Here we review the characterization of endophenotypes in a selection of environmental, genetic, and behavioral rodent models of ASD. In addition to exhibiting core ASD-like behaviors, each of these animal models display one or more endophenotypes relevant to comorbid features including altered sensory processing, seizure susceptibility, anxiety-like behavior, and disturbed motor functions, suggesting that these traits are indicators of altered biological pathways in ASD. However, the study of behaviors paralleling comorbid traits in animal models of ASD is an emerging field and further research is needed to assess altered gastrointestinal function, aggression, and disorders of sleep onset across models. Future studies should include investigation of these endophenotypes in order to advance our understanding of the etiology of this complex disorder.

Studying endophenotypes in rodent models of Autism spectrum disorder (ASD) can offer insights into the heterogeneity and underlying biological causes of this complex disorder. Patients with ASD demonstrate a high degree of variability in both the severity of core diagnostic symptoms (social communication deficits alongside repetitive behaviors) and in the nature and strength of a range of associated comorbidities. If comorbid traits associated with ASD are integral to the disorder we expect that many of these traits will present in animal models. However, despite the prevalence of comorbidities in patients, studies in animal models to date have largely focused on characterizing core behavioral traits. Here we review findings from salient rodent models of ASD identifying endophenotypes that parallel core ASD deficits in combination with one or more comorbid traits commonly reported in patients.

## ASD: Comorbid Traits

Comorbid traits in ASD include seizures, heightened aggression, and anxiety disorders as well as gastrointestinal problems, altered sensory processing, motor deficits, and sleep disorders (Table 1). While treatment of these issues can significantly improve quality of life for patients and their families, the biological mechanisms underlying these symptoms and their co-expression are generally unknown in the context of ASD.

**Table 1:** **Clinical comorbidities commonly associated with ASD**.

Domain	Comorbid symptoms
Epilepsy	High prevalence of epilepsy (8–25%) and EEG abnormalities (46%) in ASD patients (Amiet et al., 2008; Parmeggiani et al., 2010; Jeste, 2011)
	High rate of treatment resistant epilepsy in idiopathic autism (34%) (Dudova et al., 2011; Sansa et al., 2011)
Heightened aggression	Approximately 70% of ASD patients exhibit aggression toward caregivers (Kanne and Mazurek, 2011)
	Reactive aggression correlates with impairments in emotional regulation in children with ASD but not in typically developing children (Pouw et al., 2013)
Anxiety	40% of ASD cases associated with at least one comorbid anxiety disorder (van Steensel et al., 2011)
Gastrointestinal disturbances	Up to 90% ASD patients have chronic GI problems, most commonly constipation, also abdominal pain, diarrhea, and bloating (Parracho et al., 2005; Ibrahim et al., 2009; Buie et al., 2010)
Sensory	Tactile: heightened sensitivity to vibration and thermal pain in palm and forearm (Blakemore et al., 2006; Cascio et al., 2008)
	Auditory: atypical change detection of auditory stimuli (Gomot et al., 2006; Kwakye et al., 2011)
	Visual: superior performance in detail oriented tasks, deficits in motion perception (Dakin and Frith, 2005; Latham et al., 2013; Robertson et al., 2013)
	Altered olfaction and taste in high-functioning ASD patients (Bennetto et al., 2007; Dudova et al., 2011)
Motor impairment	Delays in gross and fine motor domains (Jeste, 2011)
	Deficits in motor planning, coordination, and gait (Rinehart et al., 2006; Jeste, 2011)
Sleep	Sleep disturbances (quality, quantity, latency to sleep) found in 40–80% of children and adolescents with ASD (Allik et al., 2006; Malow et al., 2006; Jeste, 2011)
	Sleep onset problems and night waking common in 2- to 5-year-olds with ASD (Krakowiak et al., 2008)

*Comorbid traits observed in patients with ASD are heterogeneous and include enhanced seizure susceptibility, heightened aggression, anxiety, gastrointestinal (GI) disturbances, altered sensory and motor function, and sleep disorders*.

Current estimates for the prevalence of epilepsy in ASD patients range between 8 and 25% (Hara, 2007; Jeste, 2011; Sansa et al., 2011; Woolfenden et al., 2012). Recent meta-analysis data show that epilepsy is more common in ASD patients with an intellectual disability (21.5 vs. 8%; Woolfenden et al., 2012). When epilepsy and abnormal EEG data are compared within the general ASD population, 15% of ASD subjects have an epilepsy diagnosis whereas a larger proportion (24.6%) shows interictal epileptiform EEG abnormalities during sleep (Ekinci et al., 2010). Other reports reveal that as many as 25% of ASD patients have comorbid epilepsy, and that 45.5% show non-seizure-related EEG abnormalities (Parmeggiani et al., 2010). Furthermore, one third (34%) of patients with idiopathic ASD have treatment resistant epilepsy (Sansa et al., 2011).

Aggressive behavior and elevated anxiety are frequently reported in children and adolescents with ASD. Caregiver surveys suggest that as many as 68% of ASD patients show episodes of aggression toward them (Kanne and Mazurek, 2011). Pouw et al. (2013) found that aggression behaviors in ASD are most likely due to a relative impairment in the understanding of the emotions of others. It is also estimated that 40% of ASD patients have at least one anxiety disorder (van Steensel et al., 2011). Specific phobias, obsessive compulsive disorder, and social anxiety disorder are most frequently observed.

A significant proportion of patients with ASD also suffer from gastrointestinal problems (42–90%); with constipation, chronic diarrhea, abnormal stool patterns, and stomach cramps frequently reported (Parracho et al., 2005; Valicenti-McDermott et al., 2006; Ibrahim et al., 2009; Buie et al., 2010; Wang et al., 2011a). Alterations in gastrointestinal function in the context of ASD are thought to be linked to the effects of anxiety and thereby mediated via CNS function; however investigations into mechanisms involving the enteric nervous system have not been reported.

By far the most common changes associated with ASD are those related to sensory processing which are present in over 90% of individuals diagnosed with ASD (Leekam et al., 2007). Patients with Asperger Syndrome show significantly higher sensitivity to high frequency tactile stimuli compared to control subjects (Cascio et al., 2008). Abnormalities in tactile sensitivity, as well as hypersensitivity to hot and cold stimuli have also been reported in adults with ASD (Blakemore et al., 2006). Auditory processing deficits related to the discrimination of temporally separated tones (Kwakye et al., 2011) and impaired odor detection thresholds (Bennetto et al., 2007; Dudova et al., 2011) have been documented in patients with high-functioning autism as well as subtle impairments in identifying tastes (Bennetto et al., 2007). Interestingly, aberrant motion perception can occur alongside superior visual processing performance in detail oriented tasks, highlighting the potential complexity of sensory changes in ASD patients (reviewed in Dakin and Frith, 2005; also see Latham et al., 2013; Robertson et al., 2013).

Motor abnormalities occur in 60–80% of individuals with ASD and include hypotonia, apraxia, and subtle gait anomalies (see Geschwind, 2009 for review). Abnormal fine and gross motor function, as well as delayed motor learning, dyspraxia, and postural abnormalities are also commonly reported in ASD patients (reviewed in Jeste, 2011). Finally, difficulties initiating sleep, frequent night time waking, and insomnia are frequently reported in children with ASD (Allik et al., 2006; Malow et al., 2006; Krakowiak et al., 2008; Jeste, 2011).

The systematic analysis of traits in animal models corresponding to patient comorbidities can potentially provide insight into the underlying biological mechanisms of ASD. Such outcomes may lead to the design of new therapies and benefits to patients.

## Animal Models of ASD

Over the last decade, a substantial number of rodent models of ASD have been generated (reviewed in Silverman et al., 2010a; Peca et al., 2011; Penagarikano et al., 2011; Wang et al., 2011b; Schmeisser et al., 2012; Won et al., 2012) and demonstrate face validity by replicating behavioral traits relevant to ASD. Well-characterized social and communication assessment paradigms and tests for the presence of repetitive behaviors exist for rodent models of ASD (Silverman et al., 2010a). In addition, a battery of tests is available to determine the presence of potential comorbidities including anxiety-like and aggressive behaviors, seizures, disrupted motor activity, sleep dysfunction, and sensory processing deficits (Crawley, 2007) as well as assays for gastrointestinal motility dysfunction (Roberts et al., 2007) in these models. Here we outline findings derived from investigations using these tests (Table 2) and highlight areas requiring further research (Table 3).

**Table 2:** **Endophenotypes identified in rodent models relevant to comorbid features of ASD**.

Domain	Model	Behavior
Seizure susceptibility	VPA	↑ Sensitivity to PTZ (Sobrian and Nandedkar, 1986) and electroshock-induced seizures (Kim et al., 2011)
	PPA	↑ Susceptibility to kindling with repeated intracerebroventricular infusions (MacFabe et al., 2007)
	Shank3B^−/−^	Occasional handling-induced seizures (Peca et al., 2011)
	CNTNAP2	Handling-induced seizures common in adults (Penagarikano et al., 2011)
	FAST	↑ Sensitivity to kindling and chemoconvulsant-induced seizures (McIntyre et al., 1999; Xu et al., 2004; Gilby et al., 2005)
	EL	Handling-induced seizures (Todorova et al., 1999)
	BALB/c	↑ Audiogenic seizures (Morin et al., 1994; Banko et al., 1997)
	C58/J	↑ Sensitivity to PTZ-induced seizures (Nutt and Lister, 1988)
Aggression	Shank2^−/−^	↑ Aggression in home cages although no change in resident-intruder test (Schmeisser et al., 2012)
	FAST	↑ (Reinhart et al., 2004)
	BALB/c	↑ (Brodkin, 2007; Velez et al., 2010)
Anxiety-like behavior	VPA	↑ (Mice) (Markram et al., 2008)
	Shank3B^−/−^	↑ (Peca et al., 2011)
	Shank2^−/−^	↑ (Schmeisser et al., 2012; Won et al., 2012)
	FAST	↑ Fear-potentiated startle (Anisman et al., 2000)
	BALB/c	↑ (Brodkin, 2007)
	BTBR	↑ Under some conditions (McFarlane et al., 2008; Pobbe et al., 2011)
Gastro-intestinal disturbances	BALB/c	Altered intestinal motility compared to C57BL/6 mice in response to serotonin antagonists (Neal et al., 2009)
Sensory	VPA	↓ PPI, ↑tactile sensitivity (Schneider and Przewlocki, 2005), ↓olfactory (Schneider and Przewlocki, 2005; Roullet et al., 2010) and pain (Markram et al., 2008) sensitivity
	PPA	↓ Sensorimotor function (increased tendency to slip/fall during beam task; Shultz et al., 2009)
	NL3^R451C^	↓ Acoustic startle at high decibel levels (Chadman et al., 2008)
	Shank3B^−/−^	↓ PPI (Peca et al., 2011)
	CNTNAP2	↑ Pain and olfactory sensitivity (Penagarikano et al., 2011)
	FAST	↓ Acoustic startle (Anisman et al., 2000)
	BTBR	↓ Thermal response (Silverman et al., 2010b)
Motor	NL3^R451C^	↑ Latency to fall from rotarod (Chadman et al., 2008)
	Shank3^e4–9^	Mild motor impairments (Wang et al., 2011b)
	CNTNAP2	Slight ↑ motor coordination (↑latency to fall from rotarod Penagarikano et al., 2011)
	EL	Delays in visuomotor development (McFadyen-Leussis and Heinrichs, 2005)
Sleep	VPA	Abnormal circadian rhythms (Tsujino et al., 2007)

*Endophenotypes relevant to enhanced seizure susceptibility, altered sensory function, and anxiety-like behavior were observed across environmental, monogenetic, and phenotype first models. However, each model was assessed for only a subset of the endophenotypes listed and further research is required to clarify full endophenotypic profiles. VPA, rodents administered valproate*.

**Table 3:** **An overview of endophenotypes assayed in rodent models of ASD**.

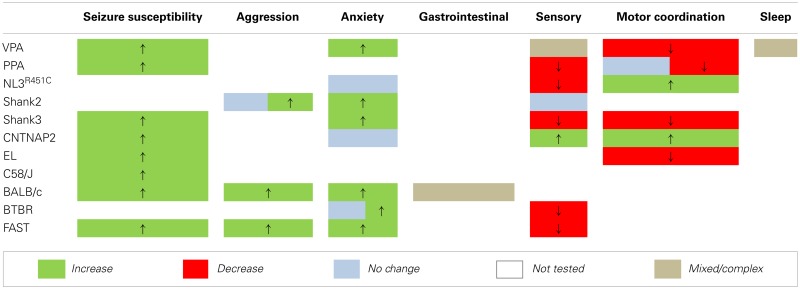

*Seizure susceptibility, sensory function, motor coordination, and anxiety-like behaviors are most commonly tested across models. Aggressive behavior, gastrointestinal function, and sleep cycles are generally understudied. Dual colored cells: formal aggression testing in Shank2^−/−^ mice did not yield data suggesting abnormal aggressive behavior, however excessive aggression was observed in home cages; PPA rats had impaired sensorimotor abilities when tested using the beam task but showed no change in swim speed in other assays. VPA, rodents administered valproate*.

Animal models are discussed in three groups; (i) models with acquired behaviors resulting from environmental insult, (ii) models expressing a human genetic mutation associated with ASD, and (iii) naturally occurring rodent strains that demonstrate behavioral endophenotypes relevant to ASD.

### Environmental models

Autism spectrum disorder-like features exhibited by environmental rodent models are generally elicited in response to an overt insult or developmental challenge, such as exposure to toxins resulting in altered neurological development.

#### Valproate models

During pregnancy, maternal exposure to the first generation antiepileptic drug valproate has been shown to significantly increase the risk of ASD in children (Rasalam et al., 2005; Meador et al., 2006; Bromley et al., 2008). Valproate is a short-chain fatty acid and is thought to reduce neuronal excitability primarily by increasing concentrations of the inhibitory neurotransmitter GABA and modulating voltage-gated sodium channels (Chapman et al., 1982; Rogawski and Loscher, 2004). In both mice and rats, exposure to valproate during gestation via intraperitoneal injection or orally with food produces deficits in social interaction and repetitive behaviors (Schneider and Przewlocki, 2005; Wagner et al., 2006; Roullet et al., 2010; Kim et al., 2011). These animals also show reduced sensitivity to pain (Markram et al., 2008) and olfactory cues (Schneider and Przewlocki, 2005; Roullet et al., 2010), increased tactile sensitivity (Schneider and Przewlocki, 2005), and diminished acoustic pre-pulse inhibition, a test commonly used to index abnormalities in sensorimotor gating (Schneider and Przewlocki, 2005; Markram et al., 2008; Gandal et al., 2010; Roullet et al., 2010). Valproate-exposed adult rats show increased levels of anxiety-like behaviors (Markram et al., 2008) and a reduced threshold for electroshock (Kim et al., 2011) and pentylenetetrazole (PTZ)-induced seizures (Sobrian and Nandedkar, 1986). These rats also show altered circadian rhythms characterized by frequent arousal during the light/sleep phase (Tsujino et al., 2007; Tables 2 and 3).

#### Propionic acid model

The gut microbiota have been suggested to play a role in the etiology of ASD (Mulle et al., 2013). Potential mechanisms contributing to ASD phenotypes are unknown, however excess toxin-producing bacteria have been identified in patients with ASD (Parracho et al., 2005) and increased levels of short-chain fatty acids (such as propionic acid; PPA) produced by enteric bacteria have been studied in rats (MacFabe et al., 2007). In rodent models, administration of the endogenous short-chain fatty acids butyric acid (Thomas et al., 2010), sodium acetate (Shultz et al., 2008, 2009), and PPA directly into the cerebral ventricles produces endophenotypes relevant to ASD (MacFabe et al., 2007, 2011; Shultz et al., 2008, 2009; Thomas et al., 2010). Acute intracerebral ventricular infusion of PPA in rats reduces sociability and learning and also produces sensorimotor impairments (Shultz et al., 2009). This paradigm also results in reduced cognitive flexibility during reversal learning (MacFabe et al., 2011). Furthermore, repeated intraventricular PPA infusion leads to increased susceptibility to kindling-induced seizures and stereotypic behavior (MacFabe et al., 2007, 2011; Shultz et al., 2009; Tables 2 and 3).

A small number of ASD patients (5%) show mitochondrial dysfunction along with altered levels of various metabolites suggestive of altered fatty acid processing (Frye et al., 2013). Further investigation to assess the effects of both PPA and valproate on gastrointestinal function (i.e., following oral administration) is needed (see Table 3), as the short-chain fatty acid receptor (GPR43) expressed by some mucosal enteroendocrine cells may play a role (Karaki et al., 2006). The effects of orally administered PPA in particular would be of interest and would serve to strengthen construct validity of this model.

### Genetic models

Many gene mutations associated with ASD code for proteins involved in the formation and maintenance of synapses (Sudhof, 2008; Betancur et al., 2009; Bourgeron, 2009; Chakrabarti et al., 2009; Betancur, 2011; Geschwind, 2011). Here we review findings from monogenic mouse models expressing mutations in four genes modulating synaptic function; the neuroligin-3^R451C^ (NL3^R451C^) mice (Tabuchi et al., 2007; Chadman et al., 2008) two models expressing specific mutations in the Shank3B/ProSAP2 gene [Shank3B knockout mice and Shank3B^e4–9^ partial knockout mice (Peca et al., 2011; Wang et al., 2011b)], as well as two SHANK2 knockout models (Schmeisser et al., 2012; Won et al., 2012) and the contactin associated protein-like 2/Neurexin IV (CNTNAP2/NRXN4; Penagarikano et al., 2011) knockout mouse model (Table 2). Electrophysiological studies in these mice report altered glutamatergic and GABAergic synaptic function (Tabuchi et al., 2007; Etherton et al., 2009, 2011; Peca et al., 2011; Wang et al., 2011b; Schmeisser et al., 2012; Won et al., 2012). Each of these models also expresses strong ASD behavioral endophenotypes suggesting a role for these genes in shaping core behaviors relevant to ASD diagnosis. However, it is not well established whether these animal models replicate comorbid traits observed in patients.

#### Neuroligin-3^R451C^ mice

Neuroligins are adhesion molecules which interact with a range of post-synaptic scaffolding proteins including Shank3 and CNTNAP2 and bind to members of the presynaptic neurexin family across the synaptic cleft (Sudhof, 2008; Krueger et al., 2012; Verpelli and Sala, 2012). Mutations in the neuroligin family of post-synaptic adhesion molecules were implicated in ASD after a spontaneous point mutation in the gene encoding NL3 was identified in two brothers with ASD; one with comorbid epilepsy (Jamain et al., 2003). Mice expressing the NL3^R451C^ mutation show a subtle reduction in pup distress calls (on post-natal day 8) and reduced acoustic startle (Chadman et al., 2008). Under some conditions and on some genetic backgrounds, NL3^R451C^ mice also show impaired social interaction (Tabuchi et al., 2007; Etherton et al., 2011). Delays in meeting developmental milestones (e.g., slower righting reflexes), which may appear as motor deficits early in development, have also been observed in these mice (Chadman et al., 2008). However, adult NL3^R451C^ mice showed better motor coordination in the accelerating rotarod test compared with wild type littermates (Chadman et al., 2008).

#### Shank3-related models

The Shank (SH3 and multiple ankyrin repeat domains) gene family (also known as Proline-rich synapse-associated proteins; ProSAPs) contains three members; Shank1-3 that code for post-synaptic scaffolding proteins involved in the recruitment of several receptors and proteins (including the neuroligins and neurexins) to the excitatory post-synaptic membrane (Irie et al., 1997; Meyer et al., 2004; Baron et al., 2006; Hayashi et al., 2009; Arons et al., 2012). Rare microdeletions within the 22q13 locus (containing Shank3) are associated with intellectual disability, speech delay, and ASD (Nesslinger et al., 1994; Bonaglia et al., 2006; Durand et al., 2007). Mutations in Shank2 are also associated with ASD (Berkel et al., 2010; Kumar, 2010). Two different genetic models in which Shank3 is altered; Shank3B^−/−^ (Peca et al., 2011) and Shank3^e4–9^ (Wang et al., 2011b) in addition to two recently reported Shank2 knockout models (Schmeisser et al., 2012; Won et al., 2012) demonstrate core and comorbid traits relevant to ASD. A third model in which one full length copy of Shank3 is deleted shows core ASD endophenotypes; however the expression of secondary/comorbid features outlined here has not been investigated in these mice (Bozdagi et al., 2010). Shank3B^−/−^ mice lacking the Shank3α and β isoforms show increased repetitive behavior (self-injurious grooming) and reduced interaction with a stranger mouse as well as occasional handling-induced seizures (Peca et al., 2011 and reviewed in Herbert, 2011). Shank3^e4–9^ mice (in which exons 4–9 are deleted) show core ASD-like deficits including social impairments, repetitive behaviors, and altered communication (i.e., less complex vocalization patterns), with learning deficits and mild motor abnormalities also evident (Wang et al., 2011b). In addition to a role as a structural protein in the central nervous system, Shank3 is present at enteric nervous system synapses (Huett et al., 2009). The enteric nervous system controls gastrointestinal motility and mucous secretion and therefore gene mutations leading to changes in synaptic function (including many ASD candidate genes) may also affect gastrointestinal function (Gershon and Ratcliffe, 2004). The Shank3 mouse models of ASD are therefore excellent candidates for investigating effects of ASD-associated gene mutations on gastrointestinal motility. Shank2 knockout mice demonstrate abnormal vocal and social behaviors, and increased grooming behaviors. Hyperactivity (e.g., repetitive jumping) and anxiety-like behaviors have also been reported in these mice (Schmeisser et al., 2012; Won et al., 2012). Schmeisser et al. (2012) detected no change in aggressive behaviors in Shank2 knockout mice using a resident-intruder assay. Despite this negative result, a high level of aggression between Shank2 knockout males was observed in home cages (Schmeisser et al., 2012).

#### CNTNAP2 mice

Genetic ablation of the contactin associated protein-like 2 (CNTNAP2) gene, a member of the neurexin transmembrane protein superfamily (also known as CASPR2 and Neurexin IV), results in ASD-like deficits in social interaction and stereotypic behaviors in mice (Penagarikano et al., 2011). In addition, CNTNAP2 knockout mice show hyperactivity, impaired nest building, and frequent handling-induced seizures after 6 months of age (Penagarikano et al., 2011). The CNTNAP2 gene has been associated with ASD and a recessive form of epilepsy (Strauss et al., 2006). These mice exhibit sensory endophenotypes including hyper-reactivity to thermal sensory stimuli and superior performance in the buried food test, an assay for olfactory function (Penagarikano et al., 2011). CNTNAP2 knockout mice also showed slightly improved motor coordination on the rotarod compared to wild type littermates. Perhaps surprisingly, the atypical antipsychotic risperidone (prescribed to treat aggression and irritability in some cases of ASD) reversed nest building deficits as well as locomotor hyperactivity in these mice (Penagarikano et al., 2011), demonstrating predictive validity in this model (Table 2).

Behavioral analyses in transgenic mouse models of ASD confirm that a range of proteins regulating synaptic function are likely to be integral to this disorder. Most studies involving genetic models have investigated one or two endophenotypes relevant to patient core and comorbid traits (Tables 2 and 3). However, to better understand the relationship between these traits a focus on assessing the more subtle secondary endophenotypes is required. Seizure susceptibility, gastrointestinal function, sleep cycles, and aggressive behaviors remain to be investigated in the majority of these genetic models of ASD (Table 3). Still, the presence of endophenotypes relevant to comorbid traits of ASD in each of these genetic models suggests that at least some of these traits may be associated with the core behavioral features of the disorder.

### Phenotype first models

Interplay between genomic and non-genomic influences (e.g., maternal effects) is almost certainly involved in the symptom heterogeneity associated with ASD. To further understand their relative degree of contribution, animal models in which clinically relevant endophenotypes occur “naturally” are of great interest. There are currently several rodent models developed via breeding processes alone that exhibit measurable endophenotypes relevant to the diagnostic criteria and comorbid traits associated with ASD. These animal models include the FAST/SLOW rats and the C58/J, BALB/c, BtBR T + tf/J (BTBR), and epileptic-like (EL) mice (Tables 2 and 3).

#### FAST/SLOW rats and EL mice

The FAST and SLOW rat strains were derived from parent populations of Long Evans Hooded and Wistar rats using selective breeding processes based on relative seizure susceptibility in the amygdala kindling model (Racine et al., 1999). This process ultimately produced a seizure-prone (FAST) and seizure-resistant (SLOW) strain. FAST rats have since proven highly seizure-prone in both the kindling model and in chemoconvulsant (e.g., pilocarpine, kainate) seizure-induction models (McIntyre et al., 1999; Xu et al., 2004; Gilby et al., 2007; Gilby and O’Brien, 2013). EL mice, like FAST rats, were also created via selective breeding based on relative seizure susceptibility and originated from the non-epileptic DDY mouse strain (Meidenbauer et al., 2011). EL mice typically exhibit handling-induced seizures by postnatal day 50–60 (Todorova et al., 1999). Remarkably, the breeding processes used to create heightened seizure sensitivity in both colonies simultaneously produced robust, comorbid ASD-like traits. Both FAST rats and EL mice exhibit significant social impairment (Reinhart et al., 2004, 2006; Gilby et al., 2007; Lim et al., 2007; Turner et al., 2007) and repetitive behaviors (e.g., overgrooming, self-injurious scratching, and/or myoclonic jumping; Gilby, 2008; Meidenbauer et al., 2011) alongside delays in social, physical, and visuomotor development (McFadyen-Leussis and Heinrichs, 2005), learning deficits, impulsivity, and hyperactivity in various testing paradigms (Anisman and McIntyre, 2002; McFadyen-Leussis and Heinrichs, 2005; Azarbar et al., 2010). FAST rats are also more aggressive than their comparison (SLOW) strain (Reinhart et al., 2004, 2006) and show reduced acoustic startle but enhanced fear conditioning (Anisman et al., 2000). Thus, FAST rats and EL mice offer a similar endophenotypic profile relevant to core and comorbid symptoms observed in ASD.

#### C58/J mice

C58/J mice naturally exhibit ASD-like traits including poor sociability (Moy et al., 2008; Ryan et al., 2010), relative learning deficits, hyperactivity (Moy et al., 2008), and stereotypic behaviors (i.e., jumping and flipping; Ryan et al., 2010). Interestingly, C58/J mice also demonstrate a reduced threshold for PTZ-induced seizures (Nutt and Lister, 1988). However, in contrast to the ASD-like developmental delays observed in FAST and EL animals, C58/J mice meet developmental milestones earlier than their comparison strain (C57BL/6J; Ryan et al., 2010).

#### BALB/c and BTBR mice

The BALB/c and BTBR mouse strains exhibit core ASD traits in the form of impaired social interaction and repetitive behaviors (i.e., overgrooming and/or excessive marble burying; Brodkin, 2007; Shoji and Kato, 2009; Pearson et al., 2011). BTBR mice also demonstrate increased social anxiety-like behavior (Pobbe et al., 2011) although anxiety responses to novel situations are inconsistent (McFarlane et al., 2008). BTBR mice are less reactive to thermal (hotplate) stimuli than the C57Bl/6J standard strain (Silverman et al., 2010b), suggesting subtle sensory changes exist in this model. In addition, several BALB/c substrains displaying distinct behavioral phenotypes offer particular strengths for comorbidity investigation. BALB/cJ mice exhibit altered gastrointestinal function (Neal et al., 2009) and are highly aggressive (Velez et al., 2010) while the epilepsy-prone (EP) BALB/c substrain is susceptible to audiogenic seizures (Morin et al., 1994; Banko et al., 1997). Notably, BTBR and BALB models have a high incidence of corpus callosal agenesis and severely reduced hippocampal commissural volumes (Wahlsten et al., 2003), which may be relevant to reports of reduced corpus callosal volumes in ASD patients (Anderson et al., 2011).

The characterization of ASD-relevant traits in these “natural” models is a relatively new initiative. Still, the documented commonalities thus far are striking; particularly the co-expression of repetitive behaviors and impaired social interaction together with heightened seizure sensitivity (Table 3). Finally, while we are aware that a few studies have investigated aggression and sensory processing in these rodent models, further testing using validated assays (Silverman et al., 2010a) should be applied to fully characterize the presence of core and comorbid features in these models.

## Summary

The primary aim of this review was to compare endophenotypic clustering within a selection of animal models of ASD. Here we focus on models expressing at least two core ASD endophenotypes with additional endophenotypes relevant to comorbid traits reported in ASD patients.

### Endophenotyping: a new approach

We report that models generated via environmental insult, genetic manipulation, and selective breeding processes demonstrate a number of overlapping endophenotypes (Tables 2 and 3) relevant to both clinical comorbid (Table 1) and core traits of ASD. Detailed investigation into the more subtle endophenotypes associated with these models is a relatively novel approach. Indeed, many clinical traits highlighted here have yet to be investigated in these models or should be re-examined using consistent methodological approaches. Until then any ranking of the clinical relevance of the phenotypic profiles would be premature. Interestingly however, enhanced seizure susceptibility, altered sensory function, anxiety-like behaviors, and changes in motor coordination were the most frequently reported endophenotypes across models (Table 3). Although not routinely investigated, several of the models also showed atypical aggressive interactions (Tables 2 and 3). Despite evidence for disturbed sleep and abnormal gastrointestinal function in a significant number of ASD patients (see Table 1), to our knowledge, circadian rhythms and gastrointestinal function have only been investigated in two models; valproate-exposed rats and BALB/c mice, respectively. As discussed, gastrointestinal motility was insensitive to serotonin antagonists in BALB/c mice in comparison to a control strain (Neal et al., 2009) and valproate-treated rats showed increased arousal during sleep compared to untreated controls (Tsujino et al., 2007; Tables 2 and 3).

### Overlapping traits

The presence of both core and comorbid endophenotypes in a range of animal models suggests that at least some of these traits may be interrelated and possibly integral to the etiology of ASD. Some endophenotypes are indeed co-expressed across different model constructs (for example, seizure susceptibility is consistently increased, as are anxiety-like behaviors in examples of environmental, genetic, as well as phenotype first models; Table 3). Both environmental models (i.e., rodents administered the fatty acids valproate and PPA) and phenotype first models show heightened seizure susceptibility and anxiety-like behaviors together with sensory and motor deficits (Table 3). In contrast, genetic models show varied changes in sensory and motor domains (Table 3) for which the underlying mechanisms are unknown.

## Future Directions: Potential Mechanisms Underlying ASD Endophenotypes

Animal models are an important tool with which to tease apart the biological mechanisms underlying ASD. Given the diverse nature of ASD, it is unlikely that a single cause is responsible for this disorder and more recent research suggests some degree of interaction between the CNS and peripheral systems. Many gene mutations identified in patients with ASD affect synaptic function (Betancur et al., 2009; Bourgeron, 2009; Betancur, 2011). This supports an emerging hypothesis that ASD is primarily a disorder of neuronal communication (Grabrucker et al., 2011; Ebert and Greenberg, 2013) and we suggest that subtle changes in neural function could underlie many of the comorbid traits described here. For example, it is well established that gene mutations coding for ion channels that result in altered synaptic function in the CNS can cause seizures in patients (Helbig et al., 2008; Goldberg and Coulter, 2013). It is also important to acknowledge, however, that many neurotransmitters and receptors that regulate neuronal communication in the CNS are of functional importance in the periphery and may thereby contribute to common comorbid traits in patient subsets. For example, in the case of gastrointestinal issues, many of the synaptic genes associated with ASD including the Shanks, neurexins, and neuroligins are also expressed in the enteric nervous system (Huett et al., 2009; Raab et al., 2010; Zhang et al., 2013), which regulates gastrointestinal motility and secretion. It is, therefore, feasible that synaptic mutations may underlie gastrointestinal symptoms in at least a subset of patients with ASD (Gershon and Ratcliffe, 2004) in addition to altering neuronal communication in the CNS. Future research should explore potential neural mechanisms underlying endophenotypes, in particular, those that are currently understudied (such as gastrointestinal disorders and altered circadian rhythms) in animal models of ASD.

In summary, rigorous endophenotyping in animal models of ASD can assist in identifying the molecular mechanisms underlying these common comorbid traits. Such information may also contribute to the identification of putative patient subsets within this spectrum of disorders and the subsequent tailoring of potential therapies. However, in order to achieve these goals, a more consistent approach in the assessment and comparison of endophenotypes is needed.

## Conflict of Interest Statement

The authors declare that the research was conducted in the absence of any commercial or financial relationships that could be construed as a potential conflict of interest.
